# The bone is the major source of high circulating intact fibroblast growth factor-23 in acute murine polymicrobial sepsis induced by cecum ligation puncture

**DOI:** 10.1371/journal.pone.0251317

**Published:** 2021-05-14

**Authors:** Jessica Bayer, Ravikumar Vaghela, Susanne Drechsler, Marcin F. Osuchowski, Reinhold G. Erben, Olena Andrukhova

**Affiliations:** 1 Department of Biomedical Sciences, University of Veterinary Medicine Vienna, Vienna, Austria; 2 Ludwig Boltzmann Institute for Experimental and Clinical Traumatology in the AUVA Research Center, Vienna, Austria; National Institutes of Health, UNITED STATES

## Abstract

Fibroblast growth factor-23 (FGF23), a bone-produced hormone, plays a critical role in mineral homeostasis. Human diseases associated with excessive intact circulating FGF23 (iFGF23) result in hypophosphatemia and low vitamin D hormone in patients with normal kidney function. In addition, there is accumulating evidence linking FGF23 with inflammation. Based on these studies and the frequent observation of hypophosphatemia among septic patients, we sought to elucidate further the relationship between FGF23 and mineral homeostasis in a clinically relevant murine polymicrobial sepsis model. Medium-severity sepsis was induced by cecum ligation puncture (CLP) in adult CD-1 mice of both sexes. Healthy CD-1 mice (without CLP) were used as controls. Forty-eight hours post-CLP, spontaneous urine was collected, and serum, organs and bones were sampled at necropsy. Serum iFGF23 increased ~20-fold in CLP compared to control mice. FGF23 protein concentration was increased in the bones, but not in spleen or liver of CLP mice. Despite the ~20-fold iFGF23 increase, we did not observe any significant changes in mineral homeostasis or parathyroid hormone levels in the blood of CLP animals. Urinary excretion of phosphate, calcium, and sodium remained unchanged in male CLP mice, whereas female CLP mice exhibited lower urinary calcium excretion, relative to healthy controls. In line with renal FGF23 resistance, expression of phosphate-, calcium- and sodium-transporting proteins did not show consistent changes in the kidneys of male and female CLP mice. Renal expression of the co-receptor αKlotho was downregulated in female, but not in male CLP mice. In conclusion, our data demonstrate that the dramatic, sex-independent rise in serum iFGF23 post-CLP was mainly caused by an upregulation of FGF23 secretion in the bone. Surprisingly, the upsurge in circulating iFGF23 did not alter humoral mineral homeostasis in the acutely septic mice. Hence, the biological function of elevated FGF23 in sepsis remains unclear and warrants further studies.

## Introduction

Fibroblast growth factor 23 (FGF23) is a 32 kDa glycoprotein mainly synthesized by osteoblast and osteocytes [1–4] in response to elevated serum phosphate [5–9], calcium [8], vitamin D [6,10,11] and parathyroid hormone (PTH) [12–16]. FGF23 is indispensable to maintain systemic mineral and vitamin D homeostasis. In renal proximal tubular epithelial cells, FGF23 controls phosphate reabsorption from the urine by downregulating the cell surface expression of the sodium-phosphate co-transporters type 2a (NaPi2a) and 2c (NaPi2c), thus augmenting renal phosphate excretion [17–21]. Furthermore, FGF23 suppresses 1α- hydroxylase (CYP27B1) [17] in proximal renal tubules, the key enzyme for producing the active vitamin D hormone 1,25(OH)_2_D_3_ [22]. In distal renal tubules, FGF23 stimulates calcium reabsorption by increased trafficking of the epithelial calcium channel TRPV5 (transient receptor potential vanilloid-5) to the apical plasma membrane [23]. Moreover, FGF23 increases the membrane abundance of the sodium-chloride co-transporter NCC, one of the key molecules involved in Na^+^-reabsorption in the kidney [24].

FGF23 signaling is mediated through the binding of the hormone to the ubiquitously expressed FGF receptors (FGFR). FGFR1c is considered to be the most important mediator of FGF23 effects [4,25]. However, FGFR3c and 4 may also be involved in FGF23 signaling in the kidney [26–28]. Under physiological conditions, the type 1 transmembrane protein αKlotho [29] serves as an essential co-receptor, allowing FGF23-mediated signal transduction by enhancing the binding of FGF23 to FGF receptors [25,30–32]. Proteolytic cleavage of full-length transmembrane Klotho produces the soluble isoform of Klotho (sKlotho) [33–35]. Recent evidence suggests that sKlotho, similar to transmembrane Klotho, functions as co-receptor for FGF23 signaling [32].

In addition to its regulatory effects on mineral homeostasis, recent studies have uncovered a new role of FGF23 in the systemic immune response. Several studies identified FGF23 as a potent stimulator of cytokine production in inflammatory cells such as macrophages, but also in hepatocytes [9,36,37]. Vice versa, inflammatory stimuli have been shown to elevate FGF23 expression and secretion from the osseous tissue [38–40]. In addition, increased *Fgf23* mRNA expression was also detected in extra osseous-tissues, especially in the spleen in lipopolysaccharide (LPS)-treated mice [36]. In the latter study, Masuda *et al*. identified activated dendritic cells and macrophages as source of the LPS-induced rise in splenic FGF23 expression. This observation was corroborated by Bansal *et al*. who also attributed the elevation of circulating Fgf23 in LPS-treated mice to an increase in splenic *Fgf23* transcription [41]. A clinical study in patients suffering from acute kidney injury (AKI) found a positive association between sepsis severity and FGF23 serum level, underlining the potentially important role of FG23 as a putative immune-regulatory molecule in sepsis [42].

Sepsis is a life-threatening organ dysfunction caused by a dysregulation in the host response to an infection [43]. Despite improvements in medical diagnostics and interventions, sepsis remains a leading cause of death in critically ill patients worldwide [44]. Despite a decline of in-hospital mortality during recent years, mortality rates persist at unacceptably high levels, ranging 25–30% in sepsis and up to 50% in septic shock [45]. Reliable, clinically relevant animal models are of crucial importance to study the heterogeneous and complex pathophysiology of sepsis.

In the current study, we used the cecal ligation puncture (CLP) procedure to investigate further the role of FGF23 in the pathophysiology of sepsis. CLP is considered the gold standard [46,47] in sepsis modeling since the resulting polymicrobial sepsis closely recapitulates the features of human abdominal sepsis [48,49]. Using this model, we analyzed circulating intact FGF23 (iFGF23), mineral homeostasis, as well as FGF23 production in the bone, spleen and liver of male and female mice. While we found a dramatic increase in circulating iFGF23 in mice of both sexes after CLP, this change did not translate into major abnormalities of mineral homeostasis. Interestingly, FGF23 protein expression was upregulated in the bone, but not in the spleen and liver of septic mice.

## Materials and methods

### Animals

Three- to six-month-old wild-type CD-1 mice (Harlan) of both sexes were kept at 22–24°C with a 12 h/12 h light/dark cycle. Mice were fed a normal mouse chow (Abbed Lab and Vet Service, Vienna, Austria) and had access to tap water *ad libitum*. All animal studies were approved by the Viennese (Austria) legislative committee and were performed in strict accordance with guidelines for animal care and welfare (Animal Use Proposal Permission no: 343130/2013/14).

### Ethical statement

To ensure a comprehensive observation, all animals were checked by trained professionals (i.e. DVMs and/or trained personnel) to identify deteriorating animals and prevent them from suffering. All mice were monitored for clinical signs of illness and their status was evaluated using our modified mouse clinical assessment scoring system (M-CASS; relying on body temperature, fur appearance, posture, mobility, alertness, startle, and righting reflex). starting 12 h post-CLP [50]. Rectal temperature was monitored (Fluke Series II thermometer, Fluke USA) at least twice daily (or more often whenever a mouse deteriorated). Starting with CLP, all mice received continuous analgesic treatment (0.05 mg/kg buprenorphine, Bupaq®, Richter Pharma, Austria) every 6–8 h.

### Sepsis model

Animals were subjected to polymicrobial sepsis using the CLP procedure. The surgery was performed under isoflurane anesthesia with perioperative buprenorphine (0.05 mg kg^-1^) in 1 ml of Ringer’s solution. In brief, the cecum was tightly ligated below the ileo-cecal valve and was perforated twice with a 17-gauge needle at its base and apex. This constitutes a mild-to-medium severity CLP as it produced an approximate mortality of 40% in 3-month-old females and 70% in 3-month-old males subjected to CLP as second hit after trauma [51]. In the current study, 23% of animals were euthanized prior to the planned study endpoint at 48h, because they met the criteria for humane endpoints based on the above-mentioned custom-developed scoring approach [50].

After repositioning of the cecum, the abdomen was closed with single button sutures and skin with Histoacryl^®^ tissue adhesive (B. Braun, Aesculap, Germany). Animals received imipenem/cilastatin (25/25 mg kg^-1^) in Ringer’s solution starting at 2 h post-CLP. For three consecutive days, animals received an additional fluid resuscitation in combination with analgesic therapy (buprenorphine/imipenem/cilastatin) twice daily, all given subcutaneously.

For the initial time course experiment, 20 μL of blood was collected via facial vein puncture from each animal as previously described by Weixelbaumer *et al*. [52], 24 h before CLP (baseline), as well as 6 h, 24 h, and 48 h after CLP. All samples were immediately diluted 1:10 in PBS containing EDTA to prevent clotting. After centrifugation 180 μL of plasma was stored at −80°C for further analysis.

At necropsy, 48 h post-CLP, approx. 1 ml of blood was collected from *vena cava* and animals were euthanized by cervical dislocation. Urine was taken directly from the bladder during necropsy and stored at -80°C. The blood was centrifuged, and serum was stored in -80°C for subsequent analysis. Liver, spleen, kidney, and bones were collected during necropsy. Healthy CD-1 mice of both sexes without any surgery were used as controls.

### Biochemical measurements

Serum creatinine, phosphorus, sodium, and calcium, as well as urinary phosphorus, sodium, and calcium were analyzed using a Cobas c111 analyzer (Roche, Mannheim, Germany). For the time course experiment, C-terminal FGF23 and iFGF23 in plasma (diluted 1:10) was measured by ELISA (Immutopics Inc., San Clemente California, USA). For the main experiment (48 h post-CLP), intact PTH and iFGF23 in serum (for iFGF23 diluted 1:3) were determined by ELISA (Immutopics Inc., San Clemente California, USA and Kainos Laboratories, Inc., Tokyo, Japan, respectively). Absorbance was read using an Enspire 2300 multilabel reader (PerkinElmer, Massachusetts, USA). In the time course experiment, the detection limit (6 pg/mL) of the iFGF23 assay (Immutopics Inc., San Clemente California, USA) was assigned to samples below the detectable range of the assay. Serum cytokine levels were assessed by using Milliplex ® MAP Mouse cytokine/chemokine magnetic Bead Panel Assay (Merck, Darmstadt, Germany) on a Bio-Plex 200 System (Bio Rad, Hercules, USA).

### RNA isolation and quantitative RT-PCR

Snap-frozen kidneys were homogenized, and total RNA was extracted using the TRI Reagent solution (Applied Bio-systems, Thermo Fischer Scientific, Bedford, USA) and reverse transcribed into cDNA using the High Capacity cDNA Reverse Transcription Kit (Applied Biosystems, Thermo Fischer Scientific, Bedford, USA). Quantitative RT-PCR was performed on a qTOWER^3^ 84 (Analytic Jena, Jena, Germany) using EvaGreen HOT FIREPol^®^ EvaGreen^®^ qPCR Mix Plus (Solis BioDyne, Tartu, Estonia). A melting curve analysis was done for all assays. Primer sequences are available on request. Expression of target genes was normalized to the expression of the housekeeping genes *low density lipoprotein receptor-related protein associated protein* (*LRPAP1*) and *death-associated protein-3* (*DAP3*).

### Total cell membrane isolation

Whole mouse kidneys were homogenized in 20 mM Tris (pH 7.4/HCl), 5 mM MgCl_2,_ 5 mM NaH_2_PO_4_, 1 mM ethylenediamine tetra-acetic acid (EDTA, pH 8.0/NaOH), 80 mM surcrose in the presence of protease inhibitors (cOmplete™ ULTRA Tablets, Mini, EDTA-free, EASYpack, Roche, Mannheim, Germany). After sonication, samples were centrifuged for 15 min at 4,000 g. Subsequently, supernatants were centrifuged for an additional 30 min at 16.000 g. Pellets were dissolved in RIPA lysis buffer (50 mM Tris, pH 7.4, 150 mM NaCl, 1 mM EDTA, 1% Triton X-100, 1% sodium deoxycholate, 0.1% SDS) and stored at -80°C until use.

### Extraction, measurement and normalization of FGF23 protein from tissue

Femora were harvested during necropsy and bone marrow was removed by brief centrifugation. Bones were snap-frozen in liquid nitrogen and kept at -80°C until use. Bone protein was extracted as described previously [53]. In brief, bones were incubated overnight in 1.2 M HCl with moderate agitation at 4° C. Subsequently, bones were incubated for 72 h in 6 M guanidinium-HCl at 4°C while shaking. The supernatant of the latest extraction fraction was precipitated with 100% EtOH, washed with 75% EtOH, and the protein pellet was resuspended in RIPA lysis buffer (50 mM Tris, pH 7.4, 150 mM NaCl, 1 mM EDTA, 1% Triton X-100, 1% sodium deoxycholate, 0.1% SDS).

At necropsy, the liver and spleen were harvested, shock-frozen in liquid nitrogen and stored at -80°C until further analysis. Frozen organs were homogenized in RIPA lysis buffer, and used for analysis. All solutions for homogenizing and protein extraction were supplemented with protease inhibitors (cOmplete™ ULTRA Tablets, Mini, EDTA-free, EASYpack, Roche, Mannheim, Germany). Bone and soft tissue lysates were used to quantify intact FGF23 (iFGF23) protein levels with the help of an ELISA Kit (Kainos Laboratories, Inc., Tokyo, Japan). Prior experiments were conducted to verify the suitability of the lysates for ELISA measurements. To overcome the inhibitory effects of the buffer components, lysates were diluted with the ELISA Kit internal standard 1 (0 pg/ml iFGF23) before being used in the ELISA. The iFGF23 levels were normalized to the total protein amount of each sample which was determined using Pierce™ BCA Protein Assay Kit (Thermo Fischer Scientific, Waltham, USA).

### Western blot

Total cell membrane protein samples were solubilized in Laemmli buffer and heated for 10 min at 97°C. Forty μg of protein/well were electrophoretically separated on a 10% polyacrylamide gel, and transferred to a PVDF membrane (GE healthcare, Chicago, USA). Consistent protein transfer was confirmed by Ponceau S staining. The membranes were blocked with 5% (w/v) non-fat dried milk, and incubated with gentle agitation at 4°C with the primary antibodies dissolved in 2% (w/v) bovine serum albumin (BSA, Sigma Aldrich/Merck KGaA, Darmstadt, Germany). As primary antibodies, we used monoclonal mouse anti-TRPV5 (1:1,000, sc-398345, Santa Cruz Biotechnology Inc., Dallas, USA), polyclonal sheep anti-NCC (1:2,000, produced in house by Dario R. Alessi, University of Dundee, Dundee, UK), monoclonal mouse anti-NaPi2a (1:1,000, NBP2-42216, Novus Biologicals, Centennial, USA), monoclonal rat anti-human Klotho (1:500, KO603, Transgenic Inc., Fukuoka-shi, Japan), monoclonal mouse anti-GAPDH (1:500, MAB374, Merck KGaA, Darmstadt, Germany), and monoclonal mouse anti-β-actin (1:5,000, A5441, Sigma Aldrich/Merck KGaA, Darmstadt, Germany). Membranes which served as negative controls for the secondary antibody were incubated with BSA instead of the primary antibody. After washing, membranes were treated with the horseradish peroxidase-linked secondary antibodies diluted in 2.5% (w/v) non-fat dried milk for 1 h at room temperature. Specific binding was visualized by the enhanced chemiluminescence (ECL) substrate (Bio Rad, Hercules, USA). Images were captured using the Chemi Doc-It 600 Image System (UVP/Analytik Jena AG, Jena, Germany). Intensity of the protein bands was quantified by using Image Quant 5.0 software (Molecular Dynamics). The expression levels were normalized to β-actin expression. Each sample was used on two independent gels/membranes to create two technical replicates. Results from both replicates were averaged for further data analysis.

### Statistics

All data represent the mean ± standard deviation of the mean (SD). All data sets were tested for normality and distribution of variance prior to analysis. In the time course experiment, a mixed model approach with Geisser-Greenhouse correction was conducted followed by Tukey *post hoc* test to assess the differences in plasma concentrations of iFGF23, C-terminal FGF23, and the iFGF23/C-terminal FGF23 ratio versus baseline within each sex. In the main experiment, two-way analysis of variance (ANOVA) was used to assess the influence of sex and of CLP, as well as their two-way interactions. In data sets showing a significant CLP effect in two-way ANOVA, individual group comparisons (CLP vs. healthy control) were performed using Student’s t-test with Welch correction. Statistical analyses were performed using Prism 7 and Prism 8 (GraphPad Software, San Diego, USA) and SPSS Statistics 25 (IBM Corp., Armonk, NY). Values of p < 0.05 were considered statistically significant.

## Results

### Sepsis increases circulating cytokine levels in both sexes

To confirm a systemic inflammatory response in CLP animals, we first examined serum levels of the inflammatory cytokines/chemokines interleukin (IL)-1ß, IL-6, IL-10, tumor necrosis factor-α (TNF-α), and keratinocyte-derived chemokine/chemokine ligand-1 (KC/CXCL1) in control and CLP mice of both sexes (Table 1). As expected, CLP mice exhibited a robust release of inflammatory mediators; e.g., serum KC/CXCL1 was ~60-fold higher in male and female CLP mice, relative to healthy controls, 48 h post-CLP. All other pro-inflammatory cytokines also tended to be elevated but due to the high variance within the CLP groups these increases did not reach statistical significance.

**Table 1:** Inflammatory markers in the serum of control and CLP mice, 48 h post-CLP.

Parameter (pg/ml)	♀Control n = 4	♀CLP n = 12	♂Control n = 4	♂CLP n = 14	Two-way ANOVA		
					Sex	CLP	Int.
***IL-1β***	13.58 ± 5.12	17.39 ± 8.14	10.98 ± 4.87	27.39 + 15.48 *	ns	p = 0.038	ns
***IL-6***	6.51 ± 3.42	1943 ± 4333	3.96 ± 2.13	5905 ± 9251	ns	ns	ns
***IL-10***	6.62 ± 2.24	432.2 ± 873.6	9.77 ± 2.56	1247 ± 1885	ns	ns	ns
***TNF-α***	7.65 ± 2.72	34.6 ± 34.30	7.48 ± 3.00	124.9 ± 215.42	ns	ns	ns
***KC/CXCL1***	75.73 ± 53.92	5229 ± 7035*	114 ± 20.81	6893 ± 8636 *	ns	p = 0.047	ns

Data are means ± SD. IL, interleukin; KC, keratinocyte-derived chemokine; CXCL1, chemokine ligand-1; TNF, tumor necrosis factor; Int., interaction between sex and CLP.

*, P<0.05 vs. healthy controls within same sex, Student’s t-test with Welch corrections.

### Sepsis-induced elevation of serum intact FGF23 is associated with an upregulated FGF23 expression in the bone but not in other tissues

**Fig 1:**
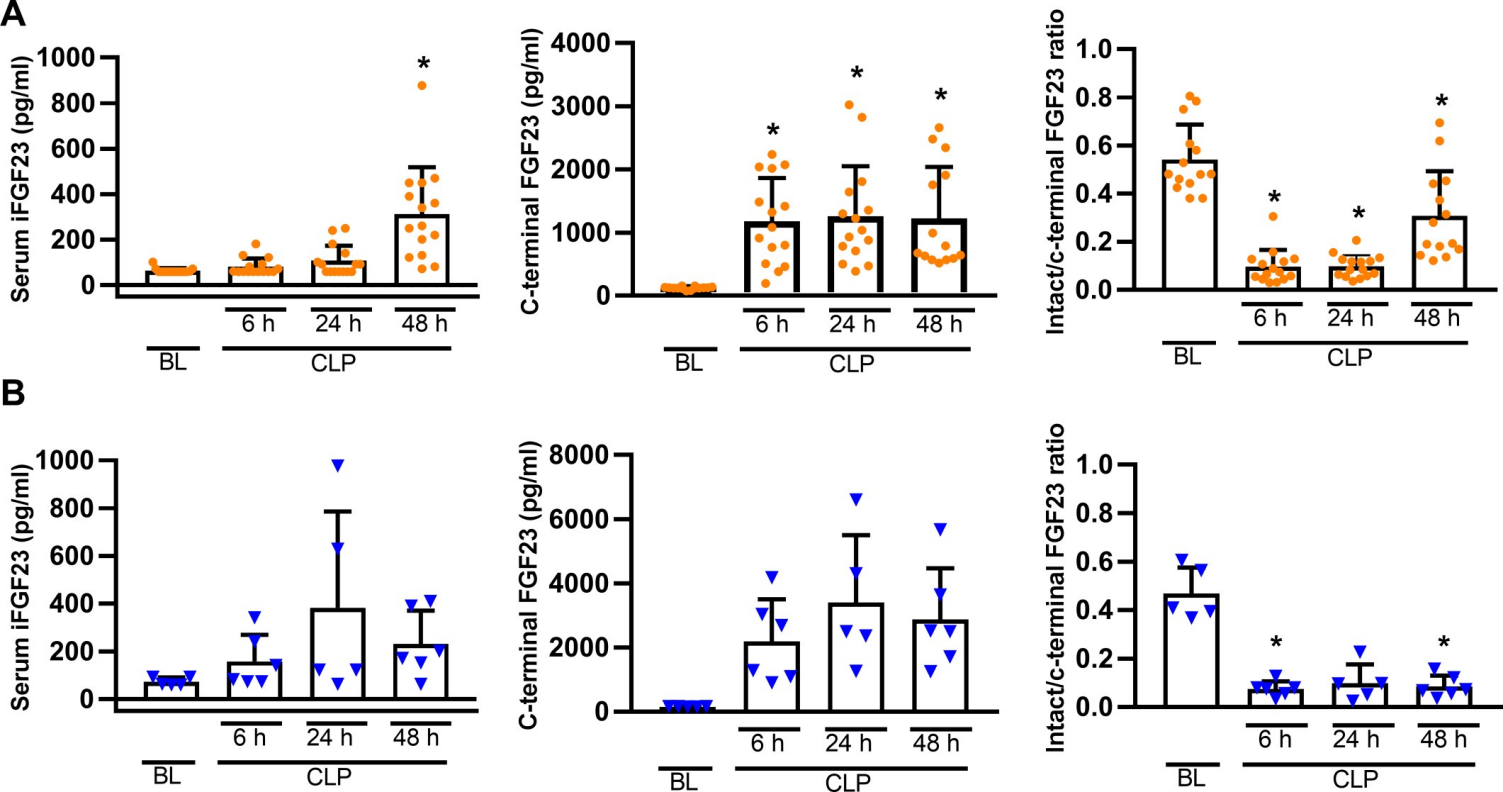
CLP-induced increase in plasma C-terminal and intact FGF23 levels. Plasma C-terminal and iFGF23 concentration in females (A) and males (B) increased post-CLP relative to baseline (BL; 24 h prior CLP). Each bar is the mean ± SD of 5–15 mice per group. Each symbol represents an individual sample. Mixed model approach with Geisser-Greenhouse correction, followed byTukey *post hoc test*.*, P<0.05 vs. baseline within same sex.

To determine the optimal time point for subsequent experiments, we first conducted a time course study evaluating the plasma concentrations of C-terminal and iFGF23 in mice of both sexes after CLP (Fig 1A and 1B). In both sexes, CLP induced a rapid and distinct increase in C-terminal FGF23 which was followed by a more delayed rise in iFG23 plasma levels. Within 6 h post-CLP, the ratio of intact to C-terminal FGF23 dropped by ~80–90% in mice of both sexes (Fig 1). Based on these data, we performed all subsequent experiments at the 48 h time point.

**Fig 2:**
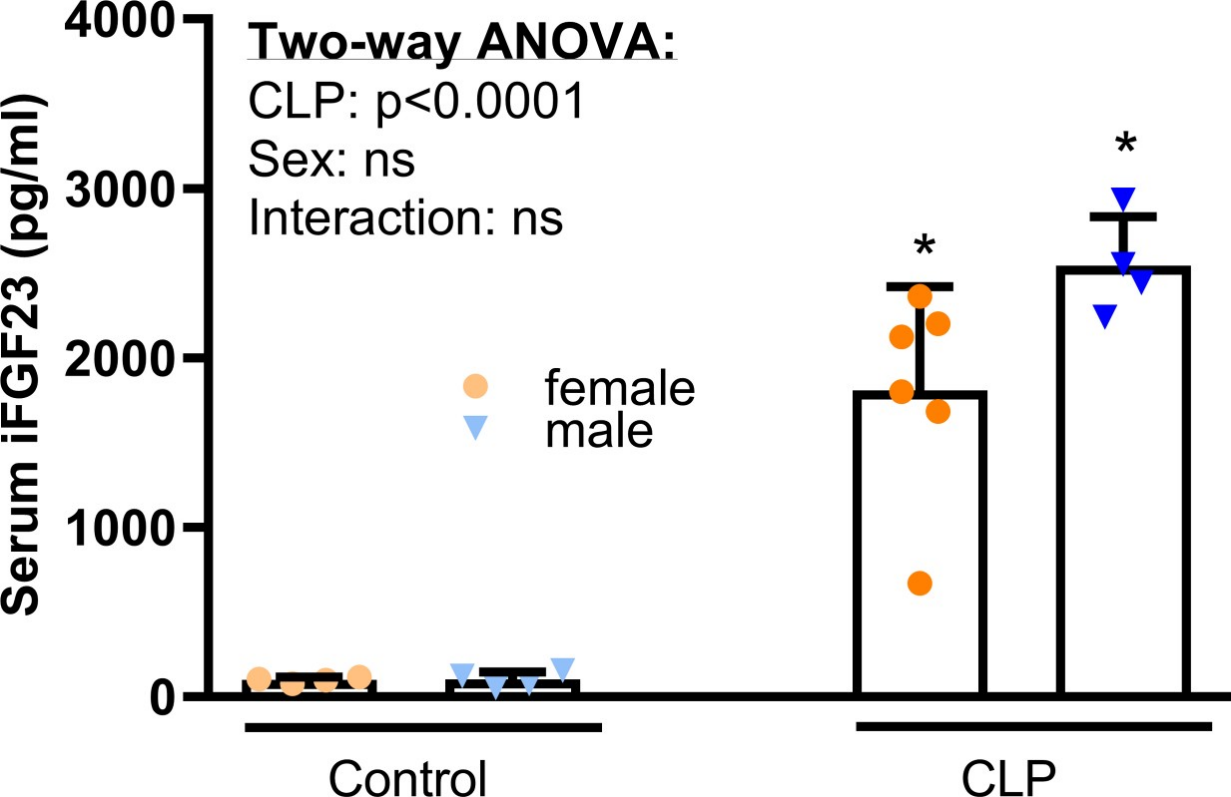
CLP-induced increase in serum iFGF23 levels, 48 h after surgery. Serum iFGF23 concentration increased profoundly in CLP mice of both sexes, relative to healthy controls. Each bar is the mean ± SD of 4–6 mice per group. Each symbol represents an individual sample. Inset shows results from two-way ANOVA. *, P<0.05 vs. healthy controls within same sex, Student’s t-test with Welch corrections.

When we measured iFGF23 serum concentration using a different assay (Kainos) compared with Fig 1 (Immutopics) in samples that were less diluted, we found the serum iFGF23 level (Fig 2) ~20-fold upregulated in CLP mice of both sexes vs. healthy controls, 48 h after CLP.

**Fig 3:**
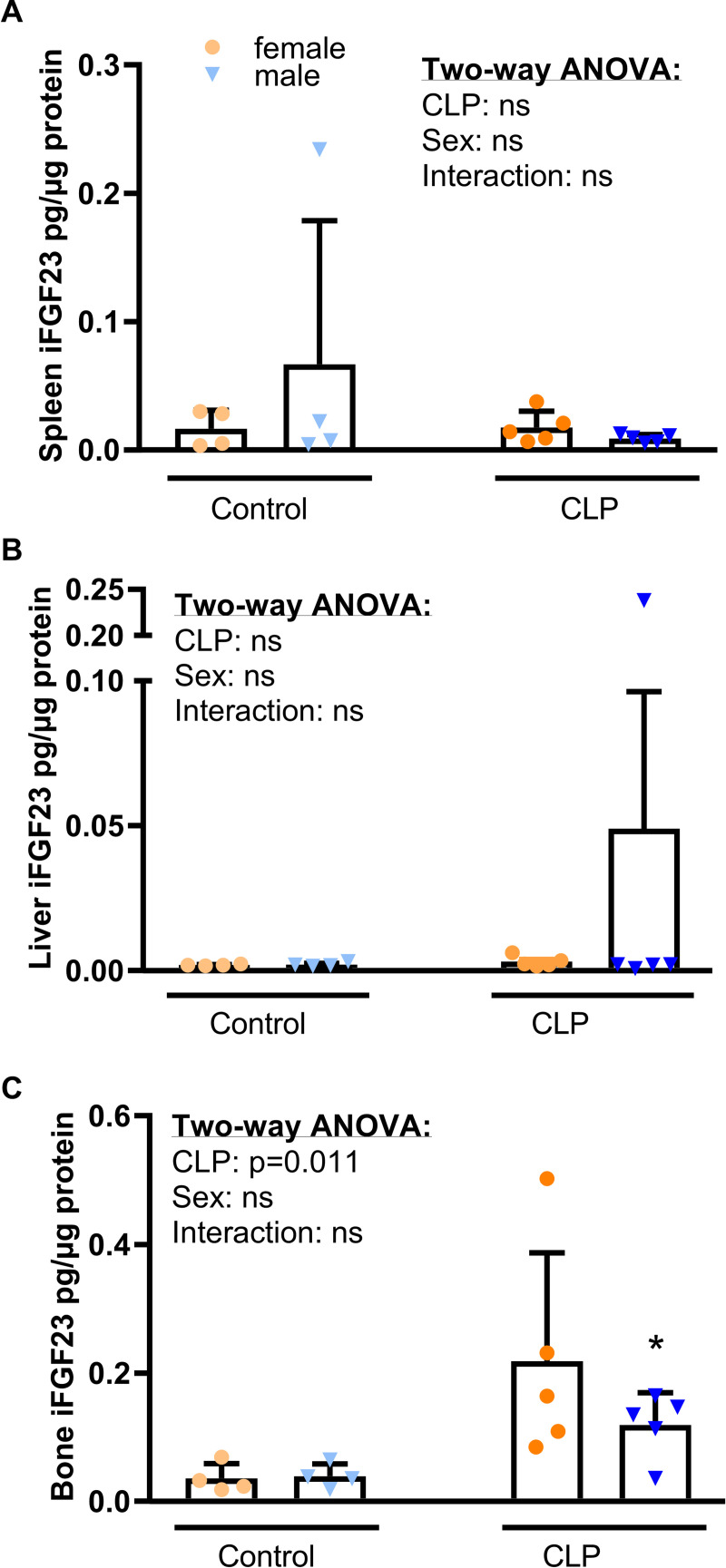
iFGF23 protein expression in spleen, liver and bone after CLP. (A) Splenic, (B) hepatic and (C) bony iFGF23 protein expression measured in lysates from spleen, liver and bone by ELISA, 48 h post-surgery (n = 4–5 per group). Data are mean ± SD. Each symbol represents an individual sample. Insets show results from two-way ANOVA. *, P < 0.05 vs. healthy controls within same sex by Student’s t-test with Welch correction.

Prior studies [9,36,41] identified the spleen, more specifically resident macrophages and dendritic cells, as the major FGF23 expression site after an inflammatory stimulus. Additionally, Masuda *et al*. reported an increased hepatic *Fgf23* expression 2 h after the injection of LPS in mice [36]. Furthermore, a clinical study with patients suffering from autosomal dominant polycystic kidney disease (ADPKD)—the most common cause of genetic CKD—showed a significantly elevated expression of hepatic FGF23, accompanied by increased circulating FGF23 [54]. To identify the major source of increased circulating FGF23 in our CLP model, we examined the abundance of intact FGF23 in spleen, liver, and bone (Fig 2). Due to the very low abundance of FGF23 protein, we were unable to quantify splenic and hepatic FGF23 protein expression, using western blotting. Therefore, we measured splenic, hepatic and bone iFGF23 protein concentrations by ELISA (Fig 3).

Interestingly, we did not observe significant differences in splenic (Fig 3A) and hepatic (Fig 3B) iFGF23 protein concentrations between control and CLP animals. In contrast, iFGF23 protein expression was markedly increased in the bones of CLP mice (Fig 3C). Hence, the bone appears to be the major source of increased circulating iFGF23 in CLP mice. Although FGF23 expression in the healthy kidney appears to be very low [17,55,56], increased renal FGF23 expression has been noted in several models of kidney disease [55,57,58]. Therefore, we quantified *Fgf23* mRNA expression in the kidney by qRT-PCR. However, *Fgf23* mRNA expression was undetectable in all groups of mice, showing that CLP does not upregulate *Fgf23* mRNA expression in the kidney (data not shown).

### Circulating PTH and mineral homeostasis remain largely unchanged in septic mice

**Fig 4:**
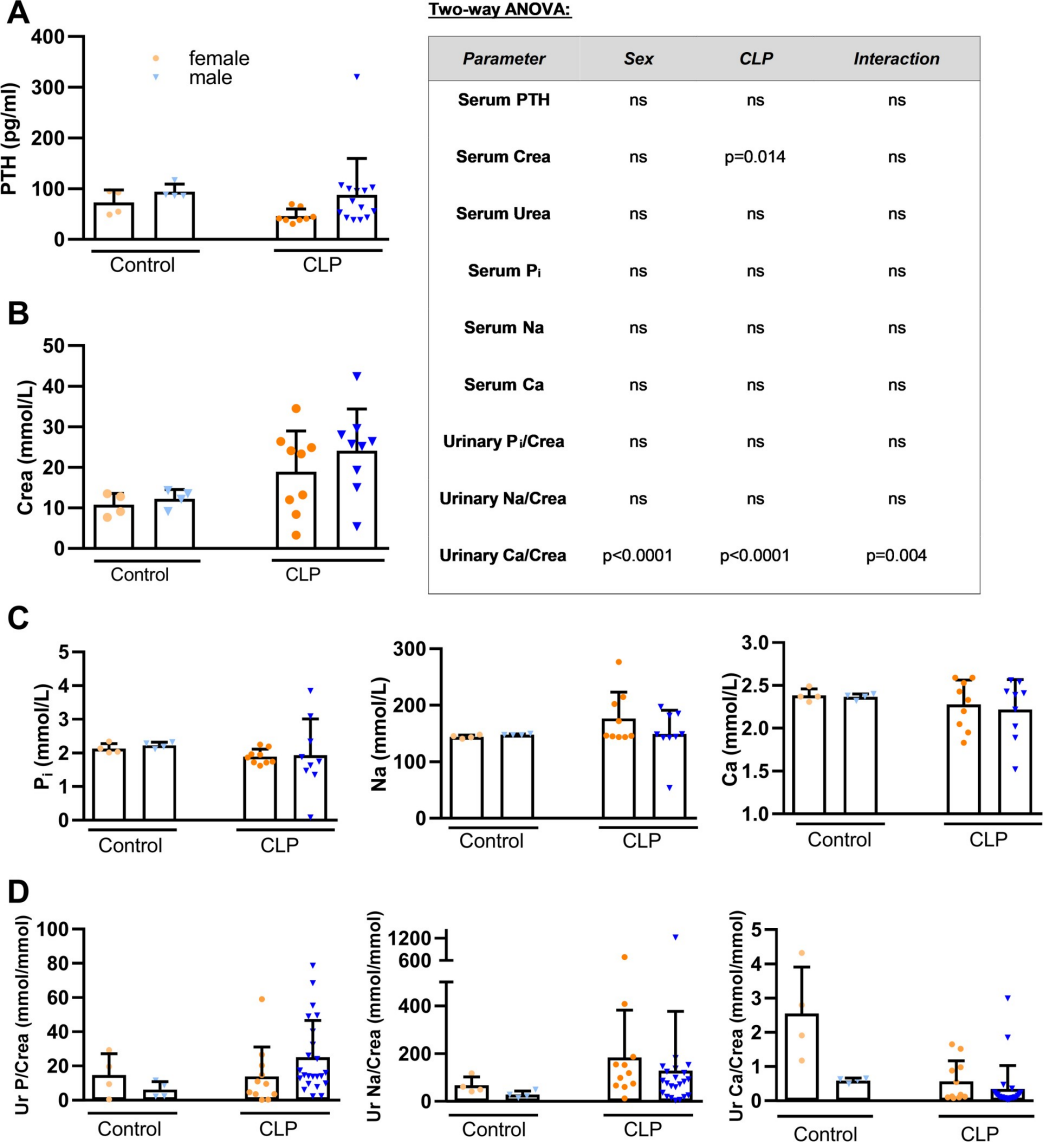
Circulating PTH concentration, serum creatinine, and mineral homeostasis in septic CLP mice. (A) Serum PTH level (healthy control female and male: n = 4 each; CLP female: n = 8, CLP male: n = 14), (B) serum creatinine (Crea), (C) serum phosphorus (P_i_), sodium (Na) and calcium (Ca) levels (n = 4–9), (D) urinary phosphorus/creatinine (Ur P/Crea), sodium/creatinine (Ur Na/Crea) and calcium/creatinine (Ur Ca/Crea) (healthy control female and male: n = 4 each, CLP female: n = 11, CLP male: n = 22–23) in control and CLP mice, 48 h post-surgery. Bars are mean ± SD. Each symbol represents an individual sample. Inset shows results from two-way ANOVA. *, P<0.05 vs. healthy controls within same sex, Student’s t-test with Welch correction.

Several studies reported that PTH acts as a stimulator of skeletal FGF23 synthesis in rodents [12–16]. In addition, a transient increase of serum PTH has been reported after administration of LPS, heat-killed *Brucella abortus*, and IL-1β in mice [40,59]. We measured serum PTH to examine its potential role as a stimulator of FGF23 secretion in septic mice. However, we observed similar PTH levels in all groups (Fig 4A).

Sepsis frequently leads to single and/or multiorgan dysfunction [60], and the kidney is one of the organs which is affected at the earliest [61,62]. To evaluate kidney function, we analyzed serum creatinine level (Fig 4B). Consistent with a declining kidney function, we found increased serum creatinine in CLP mice of both sexes. This finding is in agreement with previous studies showing elevated circulating creatinine levels already 24 h after CLP [63–65]. Despite the profound changes in circulating FGF23, serum phosphate, sodium, and calcium concentrations remained unchanged in CLP mice (Fig 4C). Similarly, with the exception of reduced urinary calcium excretion in female CLP mice (baseline vs. CLP, p = 0.056), renal excretion of phosphate and sodium was comparable in control and CLP mice (Fig 4D).

### Renal ion-transporting molecules are not regulated in a consistent manner in septic mice

**Fig 5:**
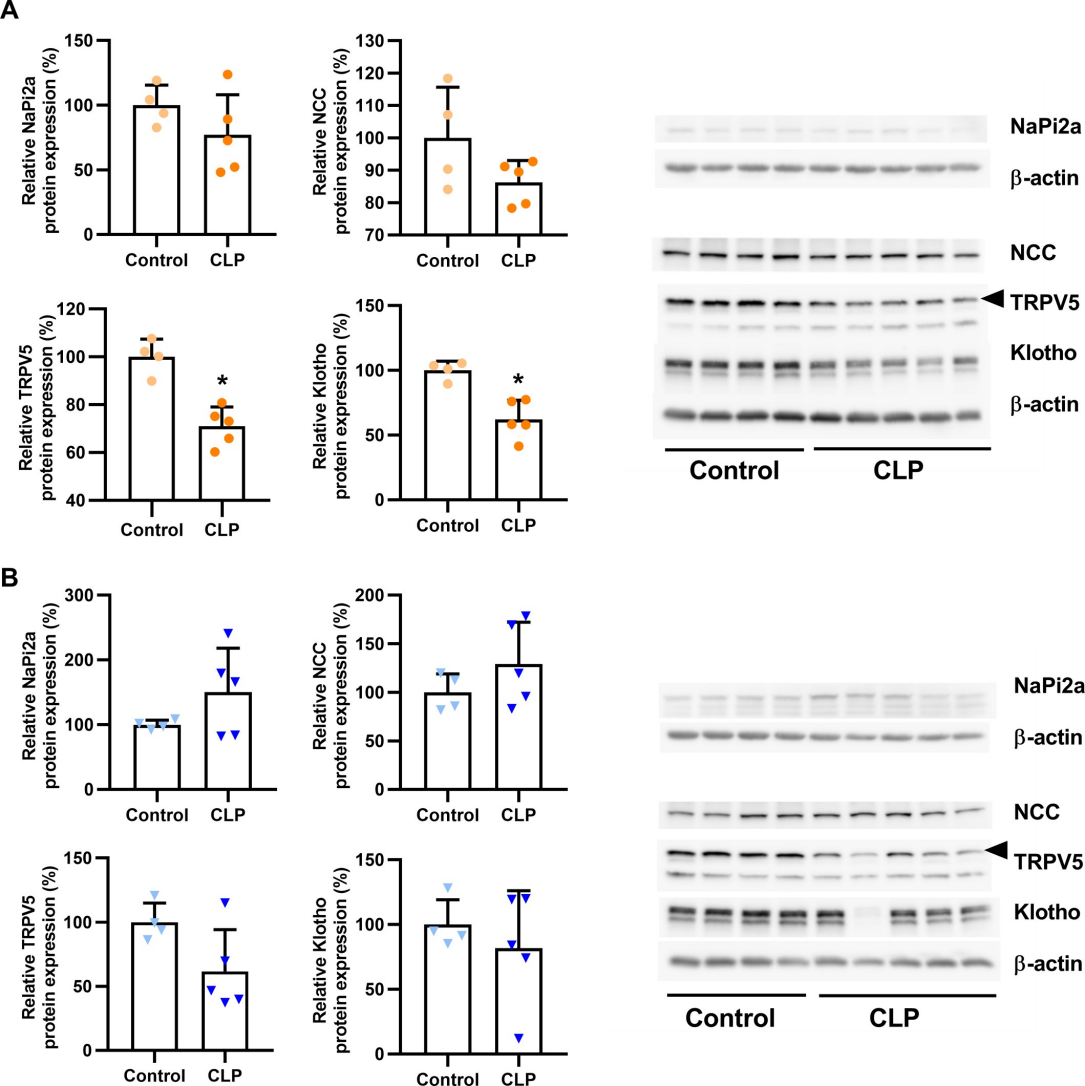
Effects of CLP-induced sepsis on the renal expression of phosphate-, sodium- and calcium-transporting proteins and of Klotho. Quantification and original western blot images of Napi2a, NCC, TRPV5 and Klotho protein expression detected in renal total membrane fractions of (A) female and (B) male healthy control and CLP mice, 48 h post-surgery. Each bar represents the mean ± SD of 4–5 mice per experimental group. Each symbol represents the mean of an individual sample measured on two membranes. *, P<0.05 vs. healthy control, Student’s t-test with Welch correction.

To further explore the puzzling finding that mineral homeostasis remained largely unchanged in CLP mice despite a distinct elevation in circulating iFGF23, we investigated typical target molecules known to be regulated by FGF23 in the kidney. To this end, we isolated membrane fractions from kidney homogenates and quantified the abundance of mineral-transporting proteins by western blotting. In female CLP mice, renal expression of NaPi2a and NCC was similar to healthy controls, whereas the expression of TRPV5 and Klotho was reduced, 48 h post-CLP (Fig 5A). In male CLP mice, NaPi2a, NCC, TRPV5 and Klotho protein expression remained unchanged relative to control mice (Fig 5B).

**Fig 6:**
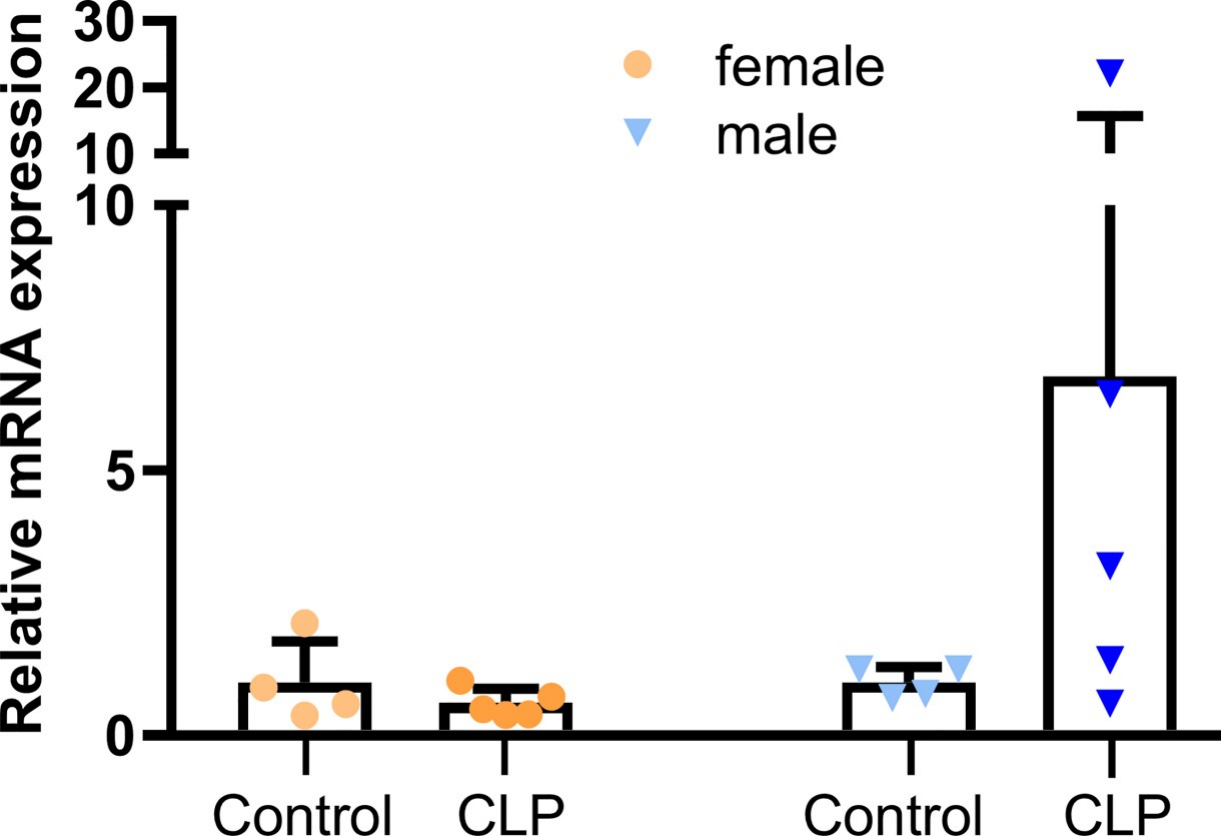
CLP-induced sepsis does not alter renal *1α-hydroxylase* mRNA expression. Relative renal mRNA expression of *1α-hydroxylase* measured by qRT-PCR in CLP mice (n = 5 female and male mice each) and healthy controls (n = 4 female and male mice each), 48 h post-surgery. Each bar represents the mean ± SD of 4–5 mice per experimental group. Each data point represents an individual animal.

FGF23 is known to regulate vitamin D metabolism in the kidney [17,56]. Therefore, we examined the mRNA expression of renal *1α-hydroxylase* (Fig 6). In female CLP mice, renal *1α-hydroxylase* remained unchanged, relative to controls. Although male CLP mice tended to have higher expression of renal *1α-hydroxylase* than healthy controls, this difference did not reach statistical significance.

## Discussion

The present study demonstrated for the first time that CLP-induced sepsis causes a profound increase in circulating intact FGF23 in mice. A number of studies reported that inflammatory stimuli induce a rise in serum FGF23 [40,41,59,66,67]. However, these studies injected either cytokines, inactivated bacteria, or LPS into mice to cause an inflammatory response. The use of LPS to investigate altered physiology during endotoxemia has been a common practice. However, LPS is a classical TLR-4 agonist and induces a rapid and transient immune response [48,68,69], which neither reflects the much milder and protracted elevation of circulating cytokines nor the hemodynamic changes occurring in human sepsis [46,70–75]. Therefore, CLP has been considered as a more appropriate recapitulation [46,47] of human abdominal sepsis including its general pathophysiology and the progressive release of cytokines in particular [48,49,69,71,76–78].

We observed that CLP-induced sepsis provoked a ~20-fold increase in circulating iFGF23 in both male and female mice, 48 h post-CLP. In agreement with the transient nature of LPS-induced endotoxemia, an FGF23 up-regulation of such magnitude and duration has never been reported in LPS-induced endotoxemia [36,41,59]. Our initial time-course experiment showed that the upregulation of circulating iFGF23 is preceded by a rapid increase in cleaved FGF23 within 6 h after CLP. The ratio of circulating intact to C-terminal FGF23, the latter encompassing both intact and cleaved FGF23, dropped precipitously within 6 h post-CLP, and remained suppressed until the 48 h time point. The latter finding suggests that CLP is associated with an increased cleavage of FGF23 and underscores the importance of measuring intact FGF23 at the protein level. The mechanisms stimulating cleavage of FGF23 in CLP mice are currently unknown and require further study.

In LPS injection models, the spleen has been identified as predominant site of FGF23 expression [36,41]. In contrast, David *et al*. postulated the bone as the main source for FGF23 synthesis during acute and chronic inflammation induced by heat-killed *Brucella abortus* or by IL-1β injection [40]. We found an upregulation of FGF23 protein only in the bone, but not in the spleen or liver of male and female CLP mice, 48 h post-CLP. In addition, *Fgf23* mRNA expression remained undetectable in the kidneys of male and female control and CLP mice. Thus, our data support the notion that the bone is the major site of FGF23 expression in CLP-induced polymicrobial sepsis.

Several clinical and experimental studies have shown a strong impact of sex and sex steroids on immune functions during physiological and pathophysiological conditions including sepsis [79–84]. Male sex and age [85] are the key risk factors for the development of sepsis [86–89]. In addition, males exhibit a higher morbidity and mortality from sepsis compared to females [90–94]. However, in our experiments, we did not observe major sex effects on cytokine or circulating iFGF23 levels in CLP mice.

FGF23 is known to be a major regulator of mineral homeostasis. FGF23 downregulates apical membrane expression of the phosphate transporters Napi2a and NaPi2c in proximal renal tubules [17–21], and upregulates the sodium- and calcium transporting molecules NCC [24] and TRPV5 in distal tubules [23]. However, despite the very high serum FGF23 level in CLP mice, renal NaPi2a expression remained unchanged in both sexes. In contrast, Ikeda *et al*. [59] as well as Meurer & Höcherl [67] found a downregulation of renal NaPi2a protein expression in LPS-treated mice, together with increased PTH secretion. David *et al*. [40] observed an increase of serum PTH levels in acute inflammation experiments. However, chronic inflammation led to a reduced PTH secretion. A cytokine-dependent regulation of PTH secretion during inflammation has been reported in several studies [95,96]. Therefore, it is possible that a rapid, pronounced cytokine release such as seen after LPS injection triggers a different PTH secretion pattern compared with CLP. In the current study, we did not observe changes in serum PTH levels, 48 h after CLP. It is conceivable that the decreased renal expression of NaPi2a in LPS-treated mice observed in the aforementioned studies are mainly due to increased PTH secretion rather than the elevated iFGF23.

In agreement with the unchanged serum concentration of sodium, CLP mice of both sexes did not show altered renal NCC protein expression compared to control mice in our study. Similarly, Olesen *et al*. [97] found unchanged expression of NCC in LPS-treated rats. However, the latter authors observed reduced serum sodium levels and increased urinary sodium excretion, which was accompanied by a downregulation of other sodium transporters across different renal compartments.

In our study, CLP mice, regardless of sex, exhibited an unchanged total serum calcium concentration, relative to healthy controls. In addition, CLP females but not males showed a reduced urinary calcium excretion, compared to healthy control mice. The renal expression of TRPV5 failed to provide an explanation for these findings, given that we observed a downregulation of TRPV5 abundance in septic females, but unchanged expression in septic males. Therefore, it is likely that other factors over-compensated for the reduced TRPV5 expression in female mice. A possible candidate might be TRPV6, which we did not measure. Meurer & Höcherl [67] have shown that LPS directly upregulates TRPV5 and 6 expression in primary renal epithelial cells. Therefore, it is possible that CLP differentially regulates TRPV5 and 6 in female mice. In addition, it is well known that the open probability of TRPV5 is regulated by endocrine and paracrine factors [98–100], so that the protein abundance not necessarily reflects the functional activity.

An important question in this context is why the increased circulating FGF23 did not downregulate NaPi2a, and failed to upregulate NCC and TRPV5 expression in the kidney of septic mice. In other words, why was sepsis associated with an apparent renal FGF23 resistance? It has been recently shown that LPS injection resulted in a ~90% reduction in renal function as measured by creatinine clearance in mice [67]. Hence, sepsis-induced acute kidney injury might render the kidney FGF23 resistant. In the current study, we could not assess creatinine clearance, because we collected only spontaneous urine. However, CLP was associated with increased serum creatinine, reflecting the CLP-induced decline in kidney function. FGF23 signaling requires the presence of the co-receptor Klotho [25,30–32] and there is convincing evidence that renal Klotho protein expression is reduced in septic patients with AKI [101], in septic foals [102], and in experimental sepsis models [101,103–106]. Similarly, we found diminished renal Klotho expression in female CLP mice. However, male CLP mice did not generally exhibit a significant decrease in Klotho protein abundance. Other studies using male mice showed a reduction of Klotho expression after inflammatory stimuli [101,106–108]. Therefore, we do not have a good explanation for the sex difference in Klotho expression in CLP mice. Nevertheless, reduced Klotho protein expression is one of the most likely scenarios for the renal FGF23 resistance observed in septic patients and experimental sepsis models.

In conclusion, our data demonstrate a robust increase in circulating iFGF23 in mice of both sexes in the acute phase of polymicrobial sepsis. The bone appears to be the major source of FGF23 in acute CLP sepsis. Because mineral homeostasis did not show major alterations in CLP mice, the biological function of the high circulating iFGF23 in acute sepsis remains unclear. Several lines of evidence support the role of FGF23 as an immune-regulatory molecule. It has been shown that FGF23 is able to regulate cytokine production in macrophages [9,36] and hepatocytes [37]. Furthermore, a transcriptome analysis in CKD mice revealed several FGF23-responsive pro-inflammatory pathways in the kidney, including TGF-β, TNF-α, and IL-1β signaling pathways [109]. Therefore, based on current knowledge [110,111], iFGF23 may serve as a positive feedback signal between various inflammatory processes and the bone. This warrants further studies aimed at delineating the pathophysiological role of FGF23 in sepsis.

## Supporting information

S1 Raw images (PDF)
