# Supplementary material for: The bone is the major source of high circulating intact fibroblast growth factor-23 in acute murine polymicrobial sepsis induced by cecum ligation puncture
Source: PLoS One. 2021 May 14;16(5):e0251317. doi: 10.1371/journal.pone.0251317 (PMC8121358; doi:10.1371/journal.pone.0251317)
Supplement: S1 Raw images — (PDF) [file pone.0251317.s001.pdf]

Females

Western Blot of renal total membrane fractions - Membrane 1

A) NaPi2a staining

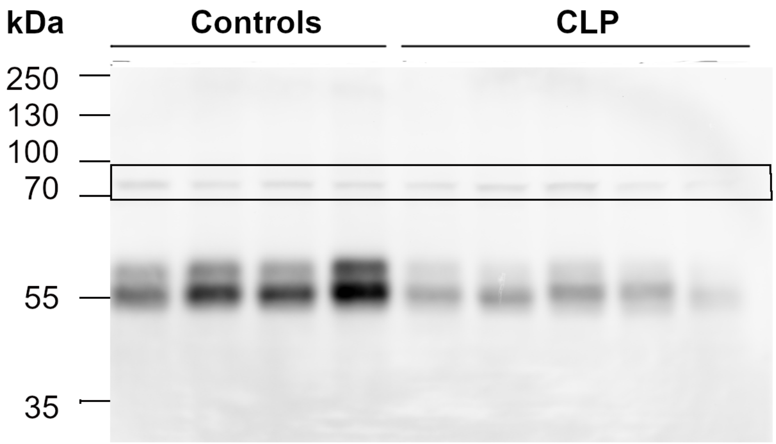

B) β-actin staining

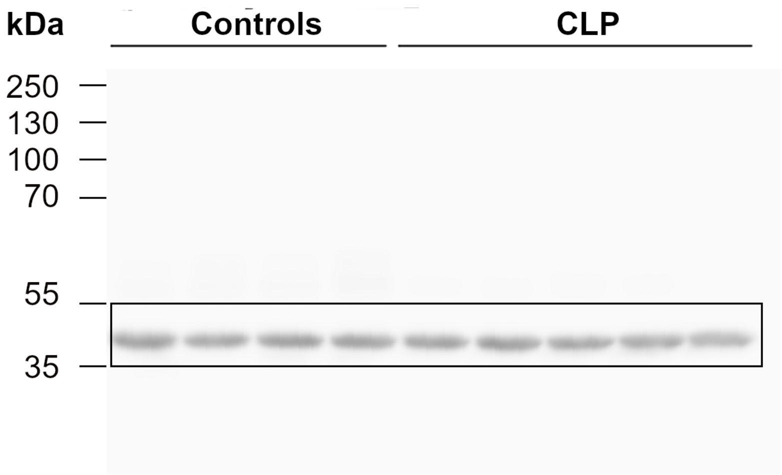

Images were captured using the Chemi Doc-It 600 Image System (UVP/Analytik Jena AG, Jena, Germany). Intensity of the protein bands was quantified by using Image Quant 5.0 software (Molecular Dynamics).
Boxes indicated respresentative picture panels for Fig 5 in the manuscript.

Females

Western Blot of renal total membrane fractions - Membrane 2

A) NaPi2a staining

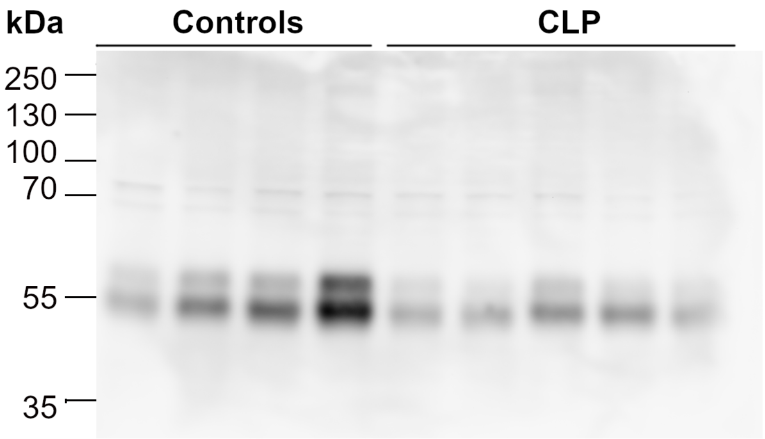

B) β-actin staining

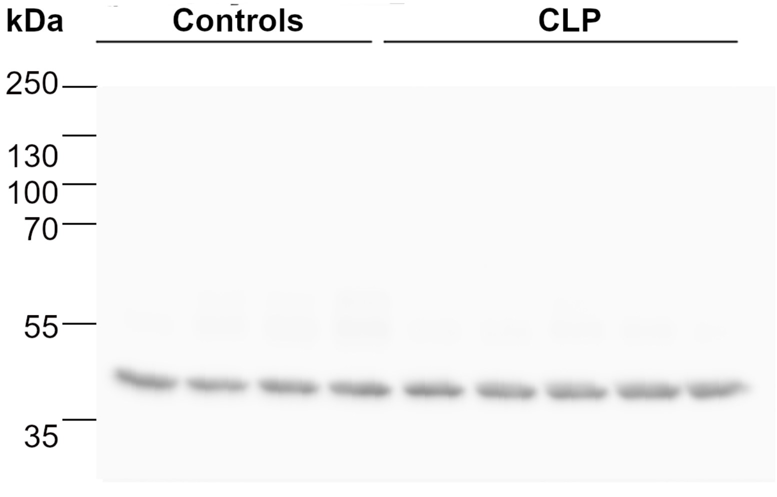

Images were captured using the Chemi Doc-It 600 Image System (UVP/Analytik Jena AG, Jena, Germany). Intensity of the protein bands was quantified by using Image Quant 5.0 software (Molecular Dynamics).

Females

Western Blot of renal total membrane fractions - Membrane 3

A) NCC staining

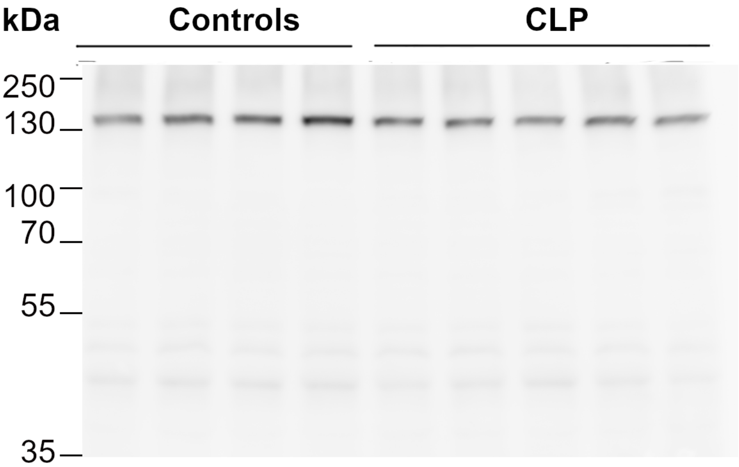

B) TRPV5 staining

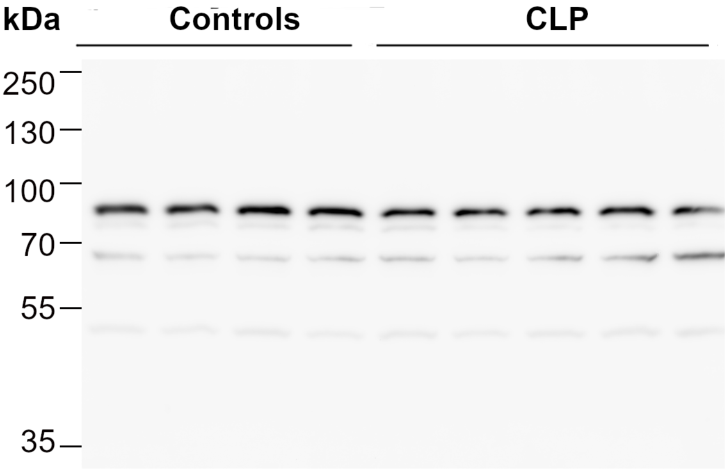

Images were captured using the Chemi Doc-It 600 Image System (UVP/Analytik Jena AG, Jena, Germany). Intensity of the protein bands was quantified by using Image Quant 5.0 software (Molecular Dynamics).

Females

Western Blot of renal total membrane fractions - Membrane 3

A) Klotho staining

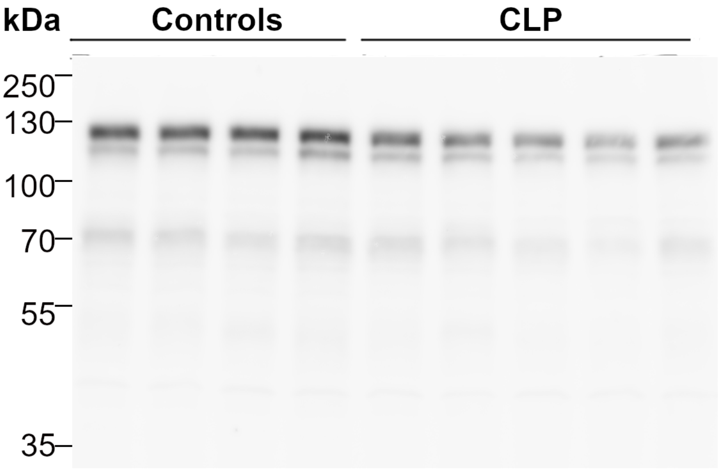

B) β-actin staining

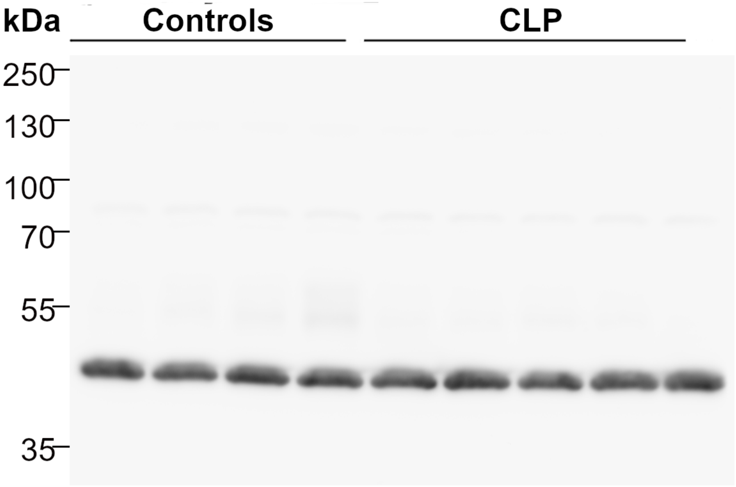

Images were captured using the Chemi Doc-It 600 Image System (UVP/Analytik Jena AG, Jena, Germany). Intensity of the protein bands was quantified by using Image Quant 5.0 software (Molecular Dynamics).

Females

Western Blot of renal total membrane fractions - Membrane 4

A) NCC staining

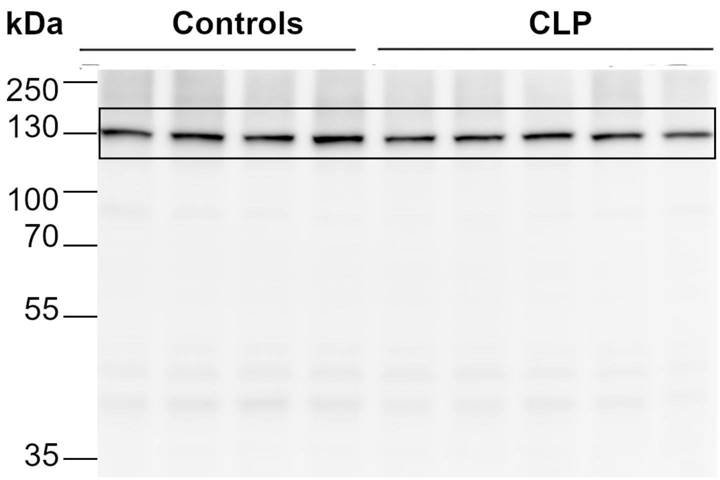

B) TRPV5 staining

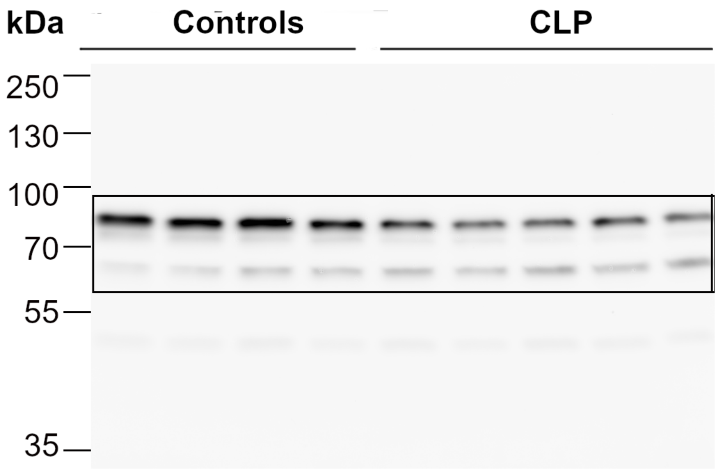

Images were captured using the Chemi Doc-It 600 Image System (UVP/Analytik Jena AG, Jena, Germany). Intensity of the protein bands was quantified by using Image Quant 5.0 software (Molecular Dynamics). Boxes indicated respresentative picture panels for Fig 5 in the manuscript.

Females

Western Blot of renal total membrane fractions - Membrane 4

A) Klotho staining

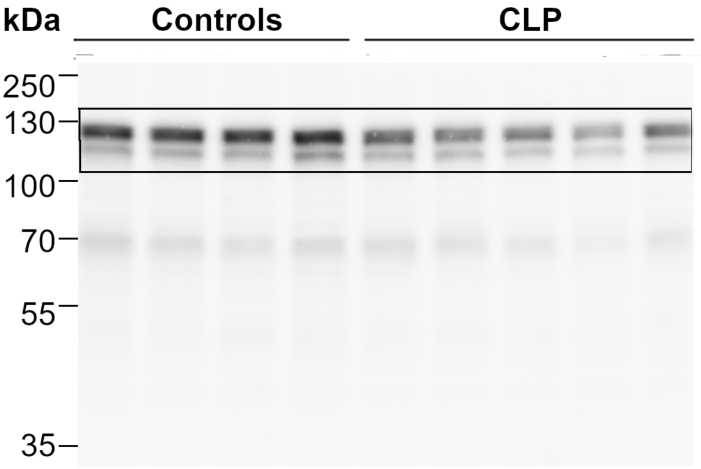

B) β-actin staining

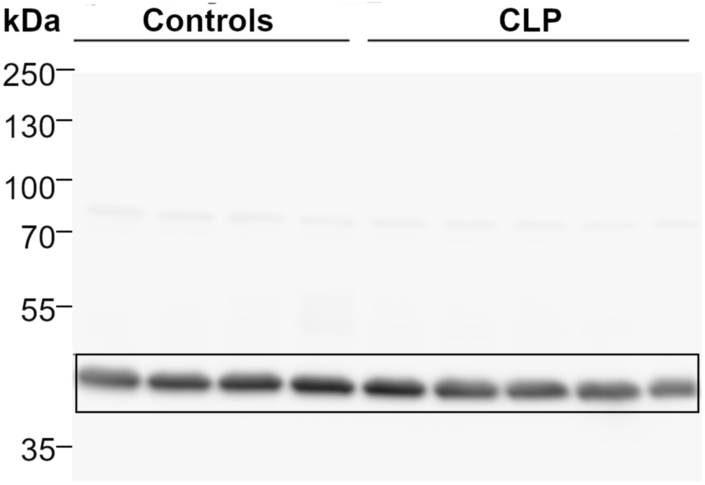

Images were captured using the Chemi Doc-It 600 Image System (UVP/Analytik Jena AG, Jena, Germany). Intensity of the protein bands was quantified by using Image Quant 5.0 software (Molecular Dynamics). Boxes indicated respresentative picture panels for Fig 5 in the manufcript.

Males

Western Blot of renal total membrane fractions - Membrane 1

A) NaPi2a staining

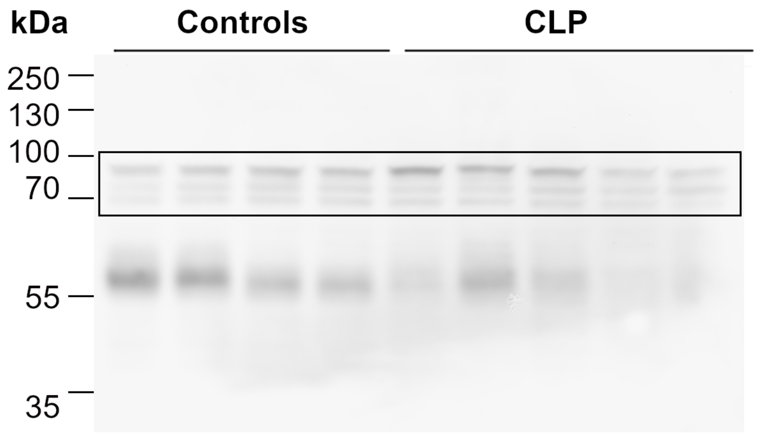

B) β-actin staining

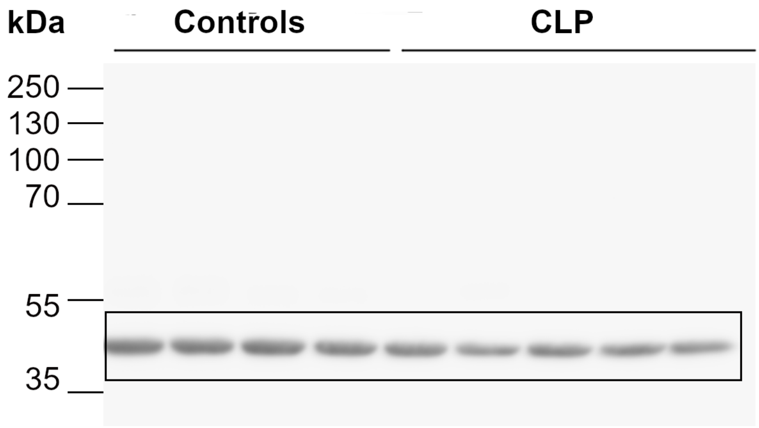

Images were captured using the Chemi Doc-It 600 Image System (UVP/Analytik Jena AG, Jena, Germany). Intensity of the protein bands was quantified by using Image Quant 5.0 software (Molecular Dynamics).
Boxes indicated respresentative picture panels for Fig 5 in the manuscript.

Males

Western Blot of renal total membrane fraction - Membrane 2

A) NaPi2a staining

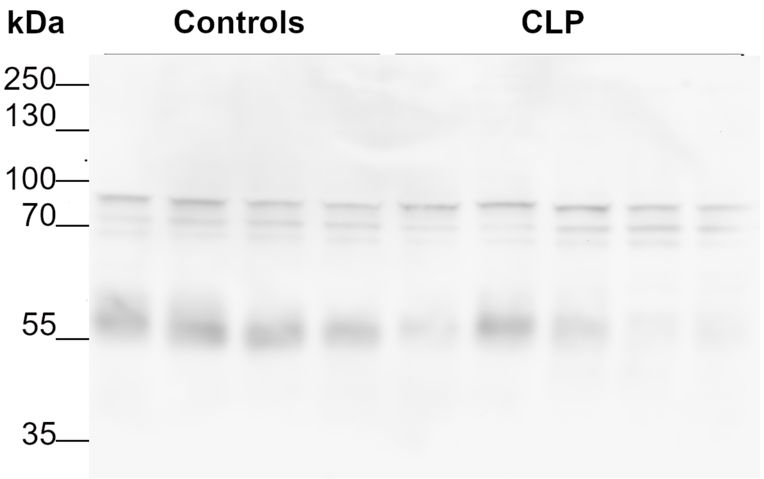

B) β-actin staining

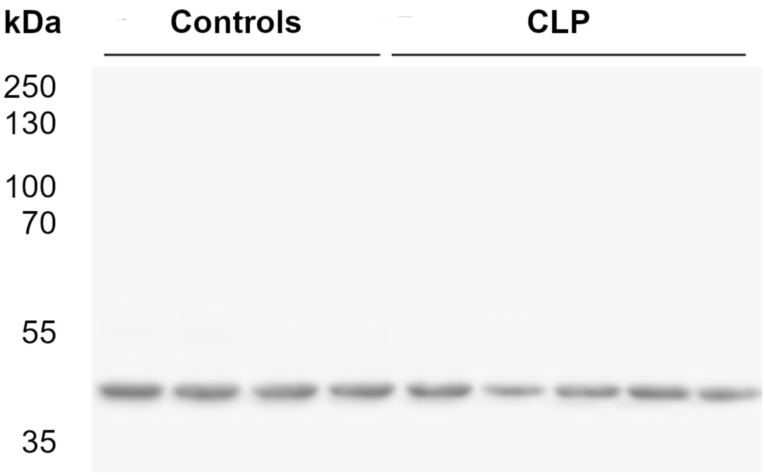

Images were captured using the Chemi Doc-It 600 Image System (UVP/Analytik Jena AG, Jena, Germany). Intensity of the protein bands was quantified by using Image Quant 5.0 software (Molecular Dynamics).

Males

Western Blot of renal total membrane fractions - Membrane 3

A) NCC staining

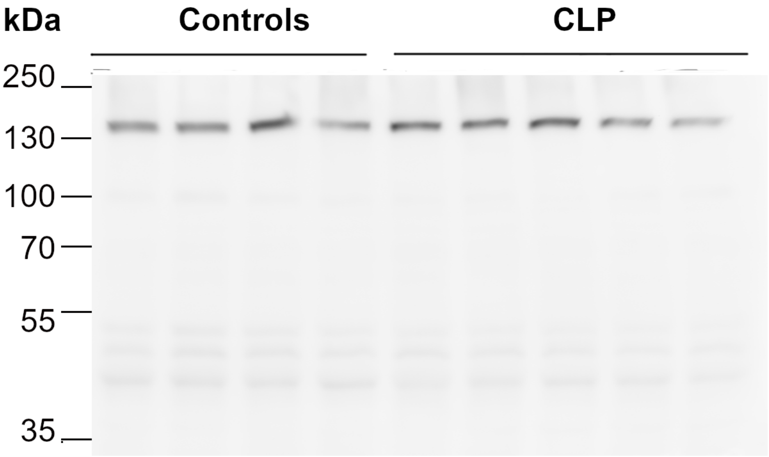

B) TRPV5 staining

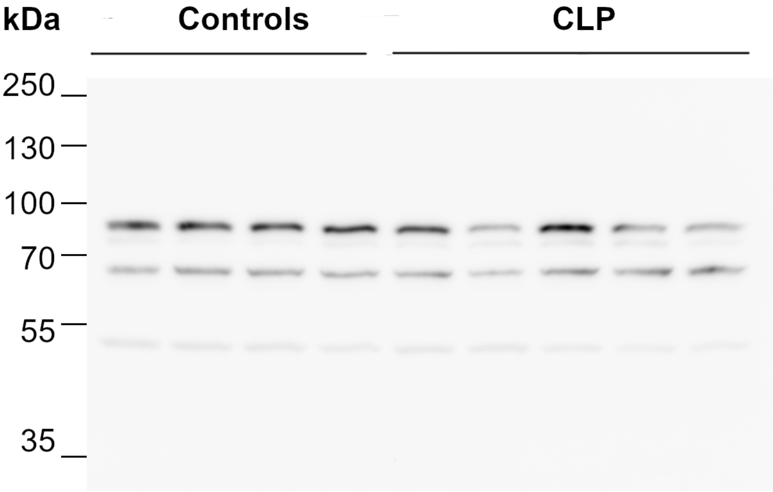

Images were captured using the Chemi Doc-It 600 Image System (UVP/Analytik Jena AG, Jena, Germany). Intensity of the protein bands was quantified by using Image Quant 5.0 software (Molecular Dynamics).

Males

Western Blot of renal total membrane fractions - Membrane 3

A) Klotho staining

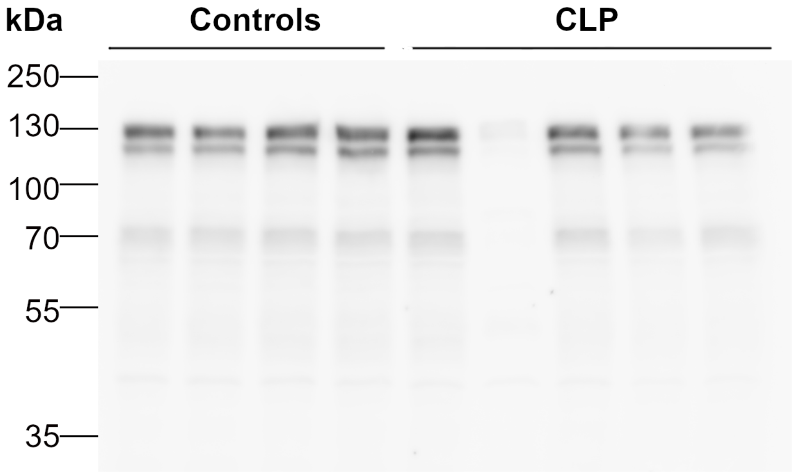

B) β-actin staining

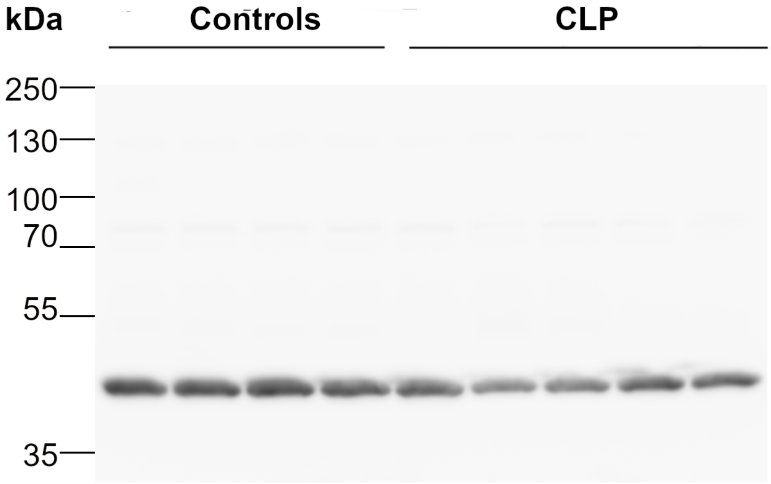

Images were captured using the Chemi Doc-It 600 Image System (UVP/Analytik Jena AG, Jena, Germany). Intensity of the protein bands was quantified by using Image Quant 5.0 software (Molecular Dynamics).

Males

Western Blot of renal total membrane fractions - Membrane 4

A) NCC staining

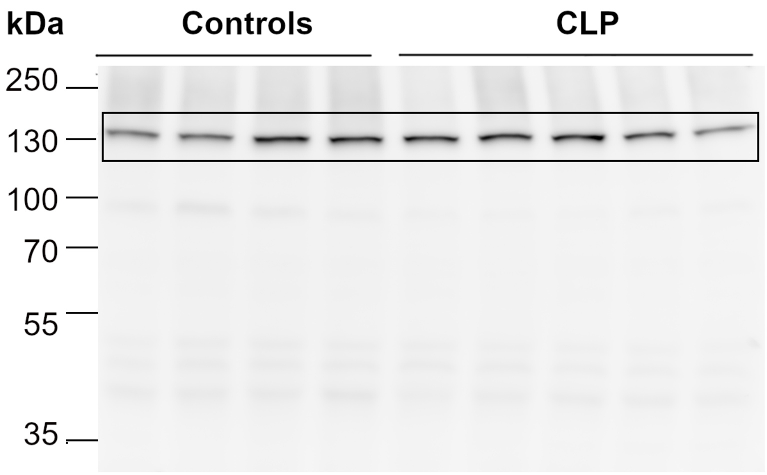

B) TRPV5 staining

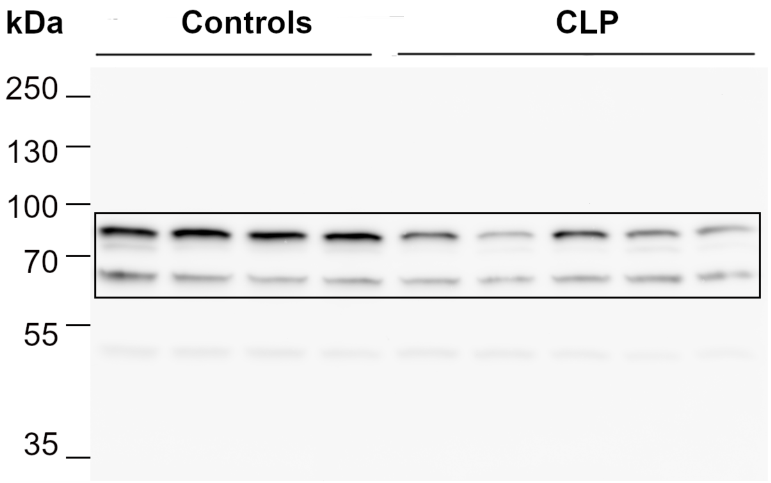

Images were captured using the Chemi Doc-It 600 Image System (UVP/Analytik Jena AG, Jena, Germany).
Intensity of the protein bands was quantified by using Image Quant 5.0 software (Molecular Dynamics).
Boxes indicated respresentative picture panels for Fg 5 in the manuscript.

Males

Western Blot of renal total membrane fractions - Membrane 4

A) Klotho staining

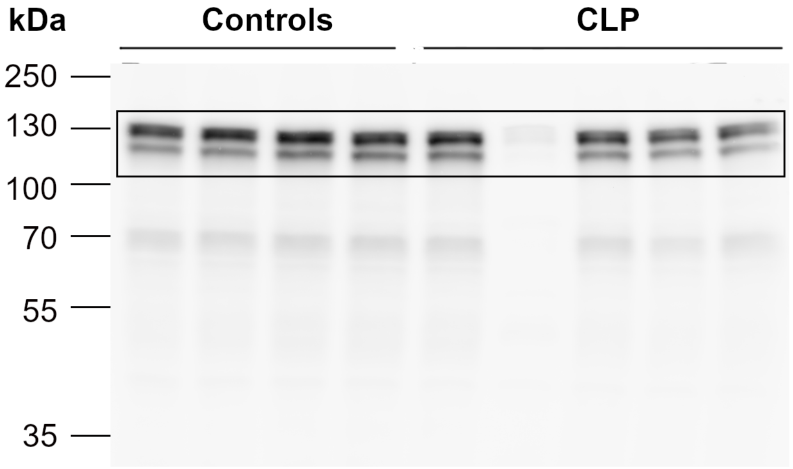

B) β-actin staining

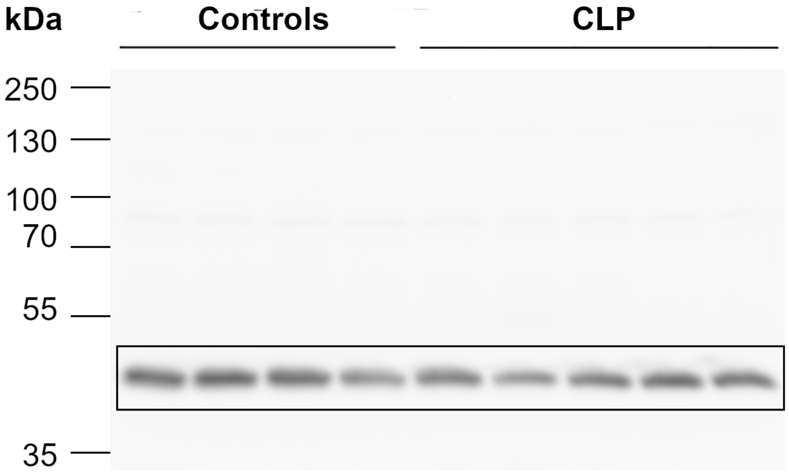

Images were captured using the Chemi Doc-It 600 Image System (UVP/Analytik Jena AG, Jena, Germany). Intensity of the protein bands was quantified by using Image Quant 5.0 software (Molecular Dynamics). Boxes indicated respresentative picture panels for Fig 5 in the manuscript.
